# Supplementary material for: A Likelihood Approach for Real-Time Calibration of Stochastic Compartmental Epidemic Models
Source: PLoS Comput Biol. 2017 Jan 17;13(1):e1005257. doi: 10.1371/journal.pcbi.1005257 (PMC5240920; doi:10.1371/journal.pcbi.1005257)
Supplement: S1 File — (TAR.GZ) [file pcbi.1005257.s014.tar.gz › HSPH_Online-SI-Revision/Additions/MSS11/n100-extreme-SEITR/n100-extreme-SEITR_NEW2_table0.pdf]

1	newly 3 weeks spec	6372.	4246.41	{4138., 4344.}	33.3583	0.176343	newly 3 weeks cumul	27 578.	19 521.5	{19 282., 19 744.}	29.2134	0.150064	newly 5 weeks spec	2747.	1742.26	{1671., 1824.}	36.5759	0.197978	newly 5 weeks cumul	34 544.	24 007.4	{23 754., 24 229.}	30.5019	0.15804	newly 51 weeks cumul	38 811.	26 847.8	{26 555., 27 073.}	30.8242	0.160058
2	newly 3 weeks spec	7321.	5197.56	{5077., 5317.}	29.0048	0.148831	newly 3 weeks cumul	36 564.	29 930.1	{29 693., 30 098.}	18.1433	0.0869518	newly 5 weeks spec	2664.	1410.57	{1349., 1468.}	47.0507	0.276387	newly 5 weeks cumul	43 633.	34 083.4	{33 866., 34 287.}	21.8863	0.107279	newly 58 weeks cumul	47 260.	35 527.2	{35 269., 35 721.}	24.8262	0.12394
3	newly 3 weeks spec	8747.	5880.86	{5749., 5970.}	32.7671	0.172589	newly 3 weeks cumul	40 258.	31 985.8	{31 654., 32 204.}	20.548	0.0999359	newly 5 weeks spec	3413.	1638.98	{1556., 1707.}	51.9783	0.319239	newly 5 weeks cumul	49 075.	36 772.1	{36 400., 36 960.}	25.0697	0.125404	newly 60 weeks cumul	54 208.	38 457.	{38 050., 38 625.}	29.0566	0.149168
4	newly 3 weeks spec	8496.	6469.25	{6358., 6566.}	23.8553	0.118415	newly 3 weeks cumul	36 998.	29 751.3	{29 508., 29 987.}	19.5868	0.0946803	newly 5 weeks spec	3304.	2437.66	{2347., 2523.}	26.2209	0.132233	newly 5 weeks cumul	45 863.	36 235.6	{35 960., 36 452.}	20.9916	0.102335	newly 58 weeks cumul	50 745.	39 752.5	{39 473., 40 039.}	21.6623	0.10604
5	newly 3 weeks spec	5120.	3327.47	{3238., 3424.}	35.0104	0.187237	newly 3 weeks cumul	31 803.	24 422.9	{24 238., 24 573.}	23.2058	0.114678	newly 5 weeks spec	1318.	676.03	{631., 717.}	48.7079	0.290509	newly 5 weeks cumul	35 687.	26 612.6	{26 390., 26 788.}	25.4278	0.12743	newly 60 weeks cumul	37 045.	27 159.6	{26 924., 27 304.}	26.685	0.134814
6	newly 3 weeks spec	4778.	3312.65	{2749., 3660.}	30.6687	0.162788	newly 3 weeks cumul	31 868.	24 039.7	{22 001., 25 307.}	24.5646	0.12324	newly 5 weeks spec	1057.	671.04	{465., 801.}	36.6036	0.209828	newly 5 weeks cumul	35 197.	26 213.4	{23 647., 27 693.}	25.5237	0.129103	newly 59 weeks cumul	35 989.	26 756.4	{24 013., 28 349.}	25.6541	0.130023
7	newly 3 weeks spec	7357.	4250.62	{4129., 4335.}	42.2235	0.23832	newly 3 weeks cumul	28 195.	18 650.1	{18 402., 18 885.}	33.8531	0.179511	newly 5 weeks spec	3977.	1866.8	{1787., 1943.}	53.0601	0.328678	newly 5 weeks cumul	37 670.	23 364.6	{23 064., 23 654.}	37.9757	0.207458	newly 51 weeks cumul	47 209.	26 672.4	{26 393., 26 996.}	43.5015	0.247981
8	newly 3 weeks spec	7216.	5273.56	{5189., 5354.}	26.9185	0.136232	newly 3 weeks cumul	32 722.	27 978.2	{27 785., 28 156.}	14.4973	0.0680265	newly 5 weeks spec	3068.	1662.91	{1583., 1728.}	45.7982	0.266235	newly 5 weeks cumul	40 537.	32 629.7	{32 427., 32 831.}	19.5064	0.0942438	newly 58 weeks cumul	46 063.	34 632.9	{34 436., 34 853.}	24.8141	0.123869
9	newly 3 weeks spec	5164.	2954.37	{2834., 3118.}	42.7891	0.242856	newly 3 weeks cumul	32 212.	24 402.4	{23 994., 25 066.}	24.2443	0.120648	newly 5 weeks spec	1330.	504.76	{449., 557.}	62.0481	0.422412	newly 5 weeks cumul	36 196.	26 143.	{25 697., 26 954.}	27.7738	0.141382	newly 60 weeks cumul	37 584.	26 498.2	{26 027., 27 357.}	29.4961	0.15187
10	newly 3 weeks spec	8694.	6501.14	{6360., 6644.}	25.2227	0.126287	newly 3 weeks cumul	33 638.	28 275.8	{28 009., 28 547.}	15.9409	0.0754272	newly 5 weeks spec	4707.	2661.07	{2572., 2739.}	43.4657	0.24782	newly 5 weeks cumul	44 803.	35 166.6	{34 901., 35 446.}	21.5083	0.105184	newly 56 weeks cumul	56 292.	39 318.6	{39 037., 39 598.}	30.1523	0.155854
11	newly 3 weeks spec	8565.	6495.57	{6360., 6633.}	24.1615	0.120159	newly 3 weeks cumul	34 772.	30 692.2	{30 472., 30 952.}	11.7329	0.054209	newly 5 weeks spec	4255.	2333.66	{2256., 2421.}	45.1549	0.26102	newly 5 weeks cumul	45 069.	36 992.4	{36 752., 37 197.}	17.9205	0.0857701	newly 55 weeks cumul	54 734.	40 137.6	{39 900., 40 330.}	26.668	0.134712
12	newly 3 weeks spec	7136.	5064.97	{4704., 5521.}	29.0223	0.149739	newly 3 weeks cumul	31 568.	25 201.2	{24 526., 26 175.}	20.1687	0.097987	newly 5 weeks spec	3139.	1812.21	{1595., 2142.}	42.2679	0.241884	newly 5 weeks cumul	39 318.	30 065.9	{28 984., 31 701.}	23.5315	0.116853	newly 56 weeks cumul	44 760.	32 577.4	{31 159., 34 881.}	27.2175	0.138541
13	newly 3 weeks spec	7637.	6298.24	{6207., 6387.}	17.5299	0.0837341	newly 3 weeks cumul	33 066.	30 046.6	{29 870., 30 202.}	9.13146	0.0415904	newly 5 weeks spec	3594.	2337.59	{2263., 2410.}	34.9585	0.186926	newly 5 weeks cumul	41 882.	36 273.	{36 121., 36 440.}	13.3924	0.0624467	newly 57 weeks cumul	49 021.	39 658.	{39 502., 39 808.}	19.1	0.0920538
14	newly 3 weeks spec	8364.	5103.94	{4994., 5215.}	38.9773	0.214573	newly 3 weeks cumul	30 226.	20 916.6	{20 575., 21 191.}	30.7992	0.159917	newly 5 weeks spec	4850.	2405.68	{2307., 2508.}	50.3984	0.304719	newly 5 weeks cumul	41 750.	26 884.2	{26 552., 27 227.}	35.6067	0.191179	newly 50 weeks cumul	54 343.	31 424.6	{31 068., 31 779.}	42.1736	0.237891
15	newly 3 weeks spec	7352.	4027.16	{3913., 4131.}	45.2236	0.261492	newly 3 weeks cumul	31 019.	20 969.8	{20 692., 21 220.}	32.397	0.17006	newly 5 weeks spec	3380.	1311.03	{1238., 1380.}	61.2121	0.411641	newly 5 weeks cumul	39 368.	24 603.6	{24 277., 24 904.}	37.5036	0.204168	newly 51 weeks cumul	45 493.	26 234.	{25 860., 26 526.}	42.3339	0.239102
16	newly 3 weeks spec	8547.	7547.09	{7460., 7626.}	11.699	0.0540542	newly 3 weeks cumul	32 656.	27 965.9	{27 821., 28 115.}	14.3621	0.0673374	newly 5 weeks spec	4015.	4410.45	{4317., 4486.}	9.84932	0.0407472	newly 5 weeks cumul	42 788.	38 233.9	{38 077., 38 369.}	10.6434	0.0488755	newly 54 weeks cumul	49 793.	50 316.	{50 210., 50 424.}	1.05037	0.00453725
17	newly 3 weeks spec	6343.	4612.14	{4154., 4862.}	27.2877	0.139142	newly 3 weeks cumul	39 322.	31 055.9	{29 744., 31 751.}	21.0216	0.102618	newly 5 weeks spec	1376.	949.89	{779., 1048.}	30.9673	0.163961	newly 5 weeks cumul	43 673.	34 131.8	{32 474., 35 000.}	21.8469	0.107236	newly 60 weeks cumul	44 678.	34 892.3	{33 047., 35 776.}	21.9028	0.107574
18	newly 3 weeks spec	6778.	4913.87	{4851., 5062.}	27.5027	0.140288	newly 3 weeks cumul	35 236.	30 161.8	{30 085., 30 501.}	14.4005	0.0676961	newly 5 weeks spec	2583.	1238.69	{1195., 1304.}	52.0445	0.320728	newly 5 weeks cumul	42 014.	33 894.6	{33 887., 34 254.}	19.3255	0.0934883	newly 59 weeks cumul	45 747.	35 083.4	{35 108., 35 474.}	23.3099	0.115518
19	newly 3 weeks spec	8875.	5717.6	{5590., 5844.}	35.5763	0.191033	newly 3 weeks cumul	35 227.	27 784.9	{27 463., 28 069.}	21.1262	0.103086	newly 5 weeks spec	4524.	1910.84	{1824., 1994.}	57.7622	0.374529	newly 5 weeks cumul	46 101.	33 059.1	{32 707., 33 401.}	28.29	0.144438	newly 54 weeks cumul	56 361.	35 431.7	{35 102., 35 761.}	37.1344	0.201605
20	newly 3 weeks spec	6233.	4549.9	{4403., 4755.}	27.003	0.136867	newly 3 weeks cumul	33 857.	27 179.2	{26 858., 27 811.}	19.7237	0.0954452	newly 5 weeks spec	2013.	1213.01	{1133., 1312.}	39.7412	0.22066	newly 5 weeks cumul	39 540.	30 767.5	{30 416., 31 646.}	22.1863	0.108989	newly 59 weeks cumul	42 220.	32 017.3	{31 605., 33 057.}	24.1655	0.12019
21	newly 3 weeks spec	6457.	4345.67	{4251., 4463.}	32.6983	0.172163	newly 3 weeks cumul	36 224.	29 342.	{29 167., 29 581.}	18.9985	0.0915296	newly 5 weeks spec	1848.	961.24	{910., 1019.}	47.9848	0.284567	newly 5 weeks cumul	41 535.	32 361.4	{32 175., 32 627.}	22.0864	0.108419	newly 60 weeks cumul	43 675.	33 176.7	{33 012., 33 475.}	24.0374	0.119438
22	newly 3 weeks spec	7395.	4940.35	{4837., 5049.}	33.1934	0.175237	newly 3 weeks cumul	32 127.	24 232.3	{24 024., 24 432.}	24.5733	0.122485	newly 5 weeks spec	2882.	1792.42	{1732., 1848.}	37.8064	0.206421	newly 5 weeks cumul	39 687.	29 021.7	{28 783., 29 263.}	26.8734	0.135934	newly 56 weeks cumul	44 378.	31 500.1	{31 248., 31 750.}	29.0186	0.148864
23	newly 3 weeks spec	4881.	4758.86	{4667., 4845.}	2.58267	0.0114081	newly 3 weeks cumul	26 601.	23 569.7	{23 354., 23 776.}	11.3953	0.052552	newly 5 weeks spec	1370.	1735.3	{1667., 1804.}	26.6642	0.102463	newly 5 weeks cumul	30 613.	28 197.7	{27 996., 28 403.}	7.88995	0.0357	newly 57 weeks cumul	31 913.	30 676.9	{30 460., 30 860.}	3.87344	0.017162
24	newly 3 weeks spec	7357.	6287.86	{6185., 6370.}	14.5323	0.0682365	newly 3 weeks cumul	40 276.	35 206.	{35 020., 35 370.}	12.5882	0.0584328	newly 5 weeks spec	2182.	1771.29	{1704., 1829.}	18.8226	0.0907601	newly 5 weeks cumul	46 540.	40 353.8	{40 168., 40 510.}	13.2923	0.0619446	newly 59 weeks cumul	48 960.	42 265.6	{42 079., 42 442.}	13.6732	0.0638571
25	newly 3 weeks spec	9005.	6759.61	{6643., 6970.}	24.9349	0.125329	newly 3 weeks cumul	36 976.	29 014.6	{28 843., 29 405.}	21.5312	0.105419	newly 5 weeks spec	3489.	2857.31	{2820., 3005.}	18.1052	0.0900565	newly 5 weeks cumul	46 402.	36 340.5	{36 302., 36 878.}	21.6834	0.106447	newly 55 weeks cumul	51 783.	40 997.8	{41 135., 41 712.}	20.8277	0.101989
26	newly 3 weeks spec	6466.	5660.79	{5558., 5772.}	12.453	0.057801	newly 3 weeks cumul	27 532.	23 235.7	{23 051., 23 377.}	15.6049	0.0736888	newly 5 weeks spec	2648.	2792.93	{2716., 2866.}	5.47319	0.0230469	newly 5 weeks cumul	34 415.	30 048.5	{29 857., 30 216.}	12.6878	0.0589295	newly 53 weeks cumul	38 357.	35 865.1	{35 587., 36 066.}	6.49649	0.0291767
27	newly 3 weeks spec	7481.	6549.33	{6461., 6658.}	12.4538	0.0577951	newly 3 weeks cumul	29 075.	25 760.9	{25 599., 25 927.}	11.3986	0.052564	newly 5 weeks spec	4125.	3516.79	{3437., 3583.}	14.7445	0.0693472	newly 5 weeks cumul	38 835.	34 130.5	{33 981., 34 270.}	12.1141	0.0560833	newly 53 weeks cumul	49 190.	42 710.1	{42 538., 42 860.}	13.1733	0.0613487
28	newly 3 weeks spec	5303.	4380.99	{4253., 4480.}	17.3866	0.0830334	newly 3 weeks cumul	34 205.	28 351.3	{28 080., 28 612.}	17.1136	0.0815304	newly 5 weeks spec	965.	992.65	{944., 1040.}	3.89534	0.0164941	newly 5 weeks cumul	37 460.	31 446.7	{31 176., 31 715.}	16.0525	0.0760039	newly 60 weeks cumul	38 276.	32 302.7	{32 045., 32 566.}	15.6058	0.0736983
29	newly 3 weeks spec	9186.	6729.86	{6613., 6865.}	26.7379	0.135168	newly 3 weeks cumul	36 850.	30 694.	{30 407., 30 945.}	16.7055	0.0793927	newly 5 weeks spec	4278.	2513.19	{2421., 2595.}	41.2532	0.23116	newly 5 weeks cumul	47 405.	37 397.4	{37 092., 37 674.}	21.1109	0.10299	newly 57 weeks cumul	55 913.	40 933.6	{40 616., 41 202.}	26.7906	0.13544
30	newly 3 weeks spec	9408.	6513.03	{6386., 6625.}	30.7714	0.159872	newly 3 weeks cumul	43 928.	36 306.7	{35 941., 36 586.}	17.3495	0.0827915	newly 5 weeks spec	3519.	1655.71	{1579., 1721.}	52.9494	0.328085	newly 5 weeks cumul	53 150.	41 306.	{40 859., 41 490.}	22.2841	0.109543	newly 60 weeks cumul	58 223.	42 857.	{42 392., 43 023.}	26.3917	0.133137
31	newly 3 weeks spec	6334.	5513.3	{5412., 5622.}	12.9571	0.0603154	newly 3 weeks cumul	24 601.	20 880.1	{20 691., 21 037.}	15.1249	0.0712277	newly 5 weeks spec	3288.	3157.54	{3082., 3236.}	4.04075	0.0179808	newly 5 weeks cumul	32 650.	28 260.4	{28 057., 28 423.}	13.4443	0.0627103	newly 50 weeks cumul	39 652.	36 688.1	{36 441., 36 925.}	7.47473	0.0337447
32	newly 3 weeks spec	8242.	6682.25	{6554., 6798.}	18.9244	0.0911495	newly 3 weeks cumul	32 101.	27 774.3	{27 571., 27 946.}	13.4785	0.0628823	newly 5 weeks spec	4308.	3116.3	{3042., 3175.}	27.6625	0.140703	newly 5 weeks cumul	42 469.	35 526.	{35 311., 35 711.}	16.3484	0.0775298	newly 56 weeks cumul	52 787.	41 377.	{41 183., 41 588.}	21.6151	0.105771
33	newly 3 weeks spec	5633.	3894.99	{3805., 3980.}	30.8541	0.160296	newly 3 weeks cumul	30 340.	23 992.3	{23 808., 24 195.}	20.9218	0.101951	newly 5 weeks spec	2012.	1010.86	{959., 1068.}	49.7584	0.299256	newly 5 weeks cumul	35 798.	27 000.6	{26 800., 27 205.}	24.5751	0.122492	newly 60 weeks cumul	38 479.	28 021.7	{27 814., 28 216.}	27.1766	0.137735
34	newly 3 weeks spec	6383.	5646.17	{5553., 5730.}	11.5436	0.0533073	newly 3 weeks cumul	29 792.	24 897.1	{24 759., 25 029.}	16.4302	0.077955	newly 5 weeks spec	2446.	2527.47	{2465., 2583.}	3.43132	0.0145856	newly 5 weeks cumul	36 225.	31 232.2	{31 071., 31 357.}	13.7828	0.0644091	newly 56 weeks cumul	39 694.	35 965.1	{35 827., 36 075.}	9.39409	0.0428453
35	newly 3 weeks spec	5611.	4210.55	{4108., 4316.}	24.959	0.124778	newly 3 weeks cumul	36 155.	28 852.1	{28 585., 29 075.}	20.199	0.098002	newly 5 weeks spec	1151.	888.74	{846., 932.}	22.7854	0.112573	newly 5 weeks cumul	39 920.	31 688.2	{31 428., 31 891.}	20.6208	0.100302	newly 60 weeks cumul	40 865.	32 424.4	{32 177., 32 643.}	20.6548	0.100488
36	newly 3 weeks spec	9648.	7590.5	{7447., 7719.}	21.3257	0.104209	newly 3 weeks cumul	37 219.	33 510.5	{33 272., 33 761.}	9.96389	0.0455897	newly 5 weeks spec	5029.	2885.31	{2810., 2964.}	42.6266	0.241395	newly 5 weeks cumul	49 093.	41 175.7	{40 899., 41 436.}	16.1271	0.0763833	newly 56 weeks cumul	60 543.	45 235.4	{44 970., 45 471.}	25.2839	0.12659
37	newly 3 weeks spec	8031.	6110.49	{5975., 6245.}	23.9137	0.118763	newly 3 weeks cumul	39 903.	34 477.8	{34 199., 34 657.}	13.596	0.0634747	newly 5 weeks spec	2840.	1647.44	{1563., 1722.}	41.9915	0.236835	newly 5 weeks cumul	47 625.	39 348.6	{39 043., 39 513.}	17.3783	0.0829166	newly 57 weeks cumul	51 821.	41 015.4	{40 745., 41 210.}	20.8518	0.101571
38	newly 3 weeks spec	6443.	3779.06	{3670., 3880.}	41.3463	0.231788	newly 3 weeks cumul	29 746.	19 872.6	{19 620., 20 122.}	33.1923	0.175196	newly 5 weeks spec	2643.	1222.39	{1162., 1270.}	53.7499	0.335146	newly 5 weeks cumul	36 633.	23 270.6	{23 005., 23 543.}	36.4764	0.197084	newly 54 weeks cumul	40 381.	24 787.5	{24 498., 25 200.}	38.6159	0.211964
39	newly 3 weeks spec	8056.	5710.66	{5503., 5947.}	29.113	0.149621	newly 3 weeks cumul	34 505.	26 966.1	{26 498., 27 530.}	21.8486	0.107106	newly 5 weeks spec	3317.	2137.79	{1988., 2335.}	35.5505	0.191612	newly 5 weeks cumul	43 007.	32 637.3	{31 992., 33 570.}	24.1117	0.119905	newly 56 weeks cumul	48 356.	35 688.3	{34 840., 37 001.}	26.1967	0.132059
40	newly 3 weeks spec	7422.	5445.07	{5347., 5548.}	26.6361	0.13456	newly 3 weeks cumul	30 770.	25 279.1	{25 070., 25 461.}	17.8449	0.0853731	newly 5 weeks spec	3674.	2132.87	{2065., 2204.}	41.9469	0.236292	newly 5 weeks cumul	39 721.	30 860.3	{30 615., 31 048.}	22.3073	0.109625	newly 57 weeks cumul	48 338.	34 132.3	{33 895., 34 315.}	29.3883	0.151128
41	newly 3 weeks spec	7711.	5187.45	{4883., 5742.}	32.7266	0.173123	newly 3 weeks cumul	45 986.	35 121.4	{34 233., 36 514.}	23.6259	0.117297	newly 5 weeks spec	1635.	997.4	{885., 1184.}	38.9969	0.217649	newly 5 weeks cumul	51 212.	38 437.4	{37 419., 40 218.}	24.9446	0.124935	newly 60 weeks cumul	52 636.	39 183.9	{38 137., 41 170.}	25.5568	0.128535
42	newly 3 weeks spec	7808.	5199.22	{5085., 5315.}	33.4116	0.176661	newly 3 weeks cumul	27 957.	20 737.3	{20 535., 20 929.}	25.8242	0.12975	newly 5 weeks spec	4650.	2667.15	{2572., 2751.}	42.6419	0.241539	newly 5 weeks cumul	38 862.	27 177.	{26 931., 27 437.}	30.0679	0.155336	newly 51 weeks cumul	51 353.	32 960.7	{32 688., 33 181.}	35.8154	0.192578
43	newly 3 weeks spec	8961.	6660.27	{6515., 6787.}	25.6749	0.128914	newly 3 weeks cumul	34 781.	28 825.4	{28 551., 29 107.}	17.1231	0.0815789	newly 5 weeks spec	4598.	2753.6	{2675., 2832.}	40.1131	0.222791	newly 5 weeks cumul	46 123.	35 932.2	{35 620., 36 262.}	22.0948	0.108443	newly 56 weeks cumul	56 656.	40 236.2	{39 933., 40 557.}	28.9815	0.148636
44	newly 3 weeks spec	7188.	5514.6	{5410., 5592.}	23.2805	0.115134	newly 3 weeks cumul	31 836.	27 101.7	{26 915., 27 275.}	14.871	0.0699281	newly 5 weeks spec	3246.	1994.66	{1932., 2063.}	38.5502	0.211668	newly 5 weeks cumul	39 959.	32 440.7	{32 239., 32 654.}	18.8151	0.0905297	newly 56 weeks cumul	46 072.	35 243.3	{35 042., 35 436.}	23.5039	0.116365
45	newly 3 weeks spec	7528.	5925.94	{5838., 6026.}	21.2813	0.10396	newly 3 weeks cumul	33 385.	29 365.2	{29 186., 29 533.}	12.0407	0.0557223	newly 5 weeks spec	3187.	2092.41	{2032., 2146.}	34.3455	0.182833	newly 5 weeks cumul	41 374.	35 000.2	{34 845., 35 153.}	15.4053	0.0726593	newly 58 weeks cumul	46 827.	37 851.3	{37 630., 38 029.}	19.1677	0.0924184
46	newly 3 weeks spec	8312.	5859.08	{5736., 5999.}	29.5106	0.151948	newly 3 weeks cumul	39 284.	32 473.5	{32 228., 32 704.}	17.3367	0.0826955	newly 5 weeks spec	3124.	1607.71	{1534., 1673.}	48.5368	0.288721	newly 5 weeks cumul	47 611.	37 195.6	{36 946., 37 402.}	21.8761	0.107223	newly 60 weeks cumul	52 255.	38 860.1	{38 608., 39 081.}	25.6337	0.128629
47	newly 3 weeks spec	8223.	4712.65	{4512., 4997.}	42.6894	0.24205	newly 3 weeks cumul	34 349.	24 107.3	{23 581., 24 990.}	29.8166	0.153859	newly 5 weeks spec	3657.	1507.31	{1411., 1633.}	58.7829	0.385588	newly 5 weeks cumul	43 642.	28 313.8	{27 721., 29 570.}	35.1227	0.188024	newly 55 weeks cumul	49 521.	30 110.8	{29 407., 31 666.}	39.1958	0.216213
48	newly 3 weeks spec	8405.	5970.12	{5819., 6158.}	28.9694	0.148658	newly 3 weeks cumul	39 417.	31 171.8	{30 834., 31 681.}	20.918	0.101941	newly 5 weeks spec	2763.	1802.74	{1709., 1893.}	34.7543	0.185857	newly 5 weeks cumul	47 174.	36 304.4	{35 935., 37 011.}	23.0416	0.113768	newly 58 weeks cumul	50 734.	38 333.3	{37 940., 39 263.}	24.4425	0.121753
49	newly 3 weeks spec	6054.	5035.06	{4955., 5114.}	16.8309	0.0800701	newly 3 weeks cumul	27 886.	22 233.7	{22 094., 22 368.}	20.2692	0.0983791	newly 5 weeks spec	2454.	2246.51	{2180., 2310.}	8.45518	0.0384809	newly 5 weeks cumul	34 341.	27 869.6	{27 704., 28 003.}	18.8444	0.0906854	newly 56 weeks cumul	38 035.	32 040.3	{31 884., 32 205.}	15.7609	0.0744898
50	newly 3 weeks spec	8264.	6962.41	{6860., 7048.}	15.7501	0.0744595	newly 3 weeks cumul	33 965.	29 463.6	{29 305., 29 596.}	13.2531	0.0617491	newly 5 weeks spec	3752.	3187.31	{3108., 3263.}	15.0504	0.0709056	newly 5 weeks cumul	43 375.	37 427.2	{37 254., 37 595.}	13.7124	0.0640544	newly 57 weeks cumul	50 179.	43 428.8	{43 245., 43 574.}	13.4523	0.0627462
